# Structural Alignment and Linguistic Contrast Help Children Learn a Key Principle of Spatial Construction

**DOI:** 10.1111/cogs.70149

**Published:** 2025-12-25

**Authors:** Yinyuan Sean Zheng, Micah Goldwater, Dedre Gentner

**Affiliations:** ^1^ Department of Psychology Northwestern University; ^2^ School of Psychology The University of Sydney

**Keywords:** Structural alignment, Contrastive comparison, Contrastive label, Spatial learning

## Abstract

Spatial representation and reasoning are important in cognition, yet they are challenging for children. Research has shown that comparison can support learning about common spatial structure and that using common labels can facilitate this process. Here, we show that a comparable pattern holds for learning about differences. That is, *contrastive* labels can promote comparison‐based learning of key spatial *differences*. In two experiments, 5‐ to 7‐year‐old children were asked to learn a key engineering principle—namely, that diagonal braces confer stability in building structures. Two factors were varied between subjects: the alignability of the training exemplars, and whether a contrastive label was used. Learning was assessed through a variety of transfer tasks, both immediately and after a delay of 2−5 days. The results showed that children in the high‐alignment condition performed better than those in the low‐alignment condition, replicating previous findings. Further, children who received the contrasting *brace* label performed better than those who did not. This suggests that hearing contrastive language can invite structural alignment and reveal differences that inform children's learning. We discuss broader implications for cognition and education.

## 1 Introduction

Spatial concepts and spatial thinking are ubiquitous, not only in direct ways such as object‐tracking or navigation, but also in abstract thinking. When we read a graph or trace through a flow chart, we are thinking in terms of space. Spatial concepts and spatial ability are critical for achieving mastery in the STEM disciplines (Science, Technology, Engineering, Mathematics). Many STEM concepts—for example, the number line, chemical structures, and static force analysis—rely on spatial depictions, and mastery of these advanced concepts hinges upon spatial skills (Cheng & Mix, 2014; Lowrie, Logan, & Ramful, 2017; Newcombe, 2010;, 2017; Stieff & Uttal, 2015). Beyond math and science, spatial metaphors pervade everyday language and cognition, as in metaphors like *love is a journey* or *conversation as tossing a ball back and forth* (Lakoff & Johnson, 1980). Cross‐linguistically, people use spatial metaphors to talk about time and other abstract domains (Boroditsky, 2000). Indeed, space has been called a “universal donor” for its role as a ubiquitous base domain for analogy (Gentner, Bowdle, Wolff, & Boronat, 2001). All of this suggests that spatial representations provide frameworks for understanding more abstract domains.

However, learning to represent and reason about space does not come easily to young children. Despite their ubiquity and perceptual availability, spatial relations are often not obvious in the world. Consider the relations of *support* and *containment*. Children are immersed in these relations from infancy, and the corresponding words (*on* and *in*) are among the most frequent words in the English language; yet, children learn these spatial prepositions later than many far less frequent nouns that label object categories (Bates et al., 1994; Frank, Braginsky, Yurovsky, & Marchman, 2021; Gentner, 1982; Gentner & Bowerman, 2009; Gleitman, Cassidy, Nappa, Papafragou, & Trueswell, 2005). Spatial concepts are challenging in part because they are inherently relational, and (unlike object concepts), difficult to individuate (Gentner & Boroditsky, 2001; Golinkoff & Hirsh‐Pasek, 2008). One indication of the challenge of learning spatial concepts is that there is considerable cross‐linguistic divergence in how languages lexicalize space (Bowerman, 1976; Bowerman & Pederson, 1992; Langacker, 1987; Levinson & Brown, 1994; Talmy, 1975). For example, children learning English must differentiate between containment events (*in*) and support events (*on*). Children learning Korean must learn a cross‐cutting differentiation between tight fit (*kkita*) and loose fit (*nehta*). For instance, a videocassette placed *in* a tight case and a ring placed *on* the finger would both be lexicalized as *kkita* in Korean (Bowerman & Choi, 2001; Choi, McDonough, Bowerman, & Mandler, 1999; Feist, 2008). Thus, each child must learn the correct way to carve up space for their language.

Children's difficulty in understanding spatial relations can also be seen in more complex tasks. For example, young children find it challenging to encode the relation of *middle*.^1^ When 3‐and 4‐year‐olds were shown that they could find a toy at the midpoint between two flags, they often failed to search at the midpoint when the same flags were placed further apart. In fact, they sometimes even searched outside of the landmarks (Simms & Gentner, 2019; see also Ankowski, Thom, Sandhofer, & Blaisdell, 2012). Children also show difficulty in map reading (DeLoache, 1989; Liben & Downs, 1989; Loewenstein & Gentner, 2001; Uttal, 2000; Yuan, Uttal, & Gentner, 2017) and in locating spatial correspondences between models (Blades & Cooke, 1994; DeLoache, 1989; DeLoache & Sharon, 2005; Loewenstein & Gentner, 2001). For example, DeLoache (1989) showed children between 33 and 35 months two rooms that differed in scale but were spatially analogous and shared perceptually similar furniture. The experimenter hid a toy in the small room and asked children to find an analogous toy “in the same place” in the bigger room. Only 37.5% of the children succeeded in finding the toy, despite having a good memory of where the original toy was hidden (83%). Further evidence suggests that children at this age rely mostly on object matches to find correspondences, rather than on recognizing the aligned spatial structure (Blades & Cooke, 1994; Gentner & Rattermann, 1991; Marzolf & DeLoache, 1994; Rattermann, Gentner, & DeLoache, 1990). For example, when asked to map between rooms that each contained a pair of identical twin objects, children below 4 responded at chance in choosing the corresponding object (Blades & Cooke, 1994). Yet, with time and experience, children come to comprehend and use spatial relations in representing and reasoning about the world. How does this learning occur?

Given the importance of spatial learning, much research has probed how children come to understand spatial relations and how to best support this learning. The current work investigates a hitherto unexplored way in which comparison and language can facilitate children's spatial learning—namely, the use of contrastive language to facilitate children's learning of key differences. Specifically, we ask whether this technique can help children learn an important but nonobvious spatial concept—that of a diagonal brace in spatial construction. Our larger agenda in this research is to explore whether hearing contrastive language can lead children to engage in their own spontaneous learning processes, lessening the need for direct pedagogical explanation. Before laying out the studies, we first review work on how comparison and language can support children's spatial learning and reasoning.

### 1.1 Comparison supports spatial learning and reasoning

One process that has been shown to aid children's relational learning is analogical comparison. This process has been widely studied under the framework of structure‐mapping theory (Falkenhainer, Forbus, & Gentner, 1989; Forbus, Ferguson, Lovett, & Gentner, 2017; Gentner, 1983, 2010; Gentner & Hoyos, 2017). According to this theory, comparison entails a process of structural alignment based on matching common relational structures, such that objects are placed into correspondence based on having like roles within the common relational structure. Achieving a structural alignment results in highlighting the common relational structure, which may be retained as a relational abstraction (Gentner, 2003; Gentner & Hoyos, 2017). Structural alignment can also result in two further outcomes: projecting one or more candidate inferences (Clement & Gentner, 1991; Falkenhainer et al., 1989; Forbus et al., 2017), and noticing alignable differences (differences that play the same role in the common structure) (Markman & Gentner, 1993a, 1996; Sagi, Gentner, & Lovett, 2012).

There is abundant evidence that comparison can render common relational structure more salient for both children and adults (Chen, 1999; Christie & Gentner, 2010; Dixon & Bangert, 2004; Doumas & Hummel, 2013; Gentner, Anggoro, & Klibanoff, 2011; Loewenstein & Gentner, 2001; Gick & Holyoak, 1983; Jee & Anggoro, 2019, 2021; Loewenstein, Thompson, & Gentner, 2003; Markman & Gentner, 1993b; Thompson & Opfer, 2010). This process can be applied to aid spatial learning. For example, Loewenstein and Gentner (2001, Expt. 2) adapted the DeLoache model‐room paradigm to examine whether comparison could help 3 ½‐year‐olds carry out a relational mapping between two model rooms.^2^ They adapted a technique used by Blades and Cooke (1994) in which both rooms had a pair of identical objects, as well as some unique items. To be correct on the unique items, the child must map (for example) chair→chair and table→table; but to succeed on the twin items, the child must use the spatial configuration—for example, front stool→front stool; back stool→back stool. An object‐mapping strategy would lead to chance performance on the twin items, even if children performed well on the unique items, as shown by Blades and Cooke (1994). To test whether comparison would enhance children's representation of the spatial relations, Loewenstein and Gentner gave half the children a close comparison task prior to the mapping task; children were given the initial model along with a nearly identical model and encouraged to compare them: for example, the experimenter pointed to two corresponding objects and said, “Do you see how these are the same?” In the control condition, children saw only one room and were asked to name the color of the furniture. Then, both groups received the standard search task. The experimenter hid a toy under an object in the model room and asked the children to find the matching toy in the “very same place” in the test room. The results showed, first, that children overall performed well on the unique objects, but failed on the twin objects—consistent with using an object‐focused strategy; and second, that comparison mattered. Children in the comparison condition were over 70% correct on the twin objects, as compared to 42% in the control group—evidence that comparison led children to attend not only to object matches but also to the aligned spatial structure. These results are also evidence that even a very close comparison can facilitate transfer to a new instance of the same relational structure—even one that differs in surface features from *both* the antecedents^3^ (Fyfe, Bishop, & Stepto, 2014; Gentner et al., 2011; Gentner, Loewenstein, & Hung, 2007; Hoyos, Horton, Simms, & Gentner, 2020; Thompson & Opfer, 2010).

In summary, a widely attested way to scaffold relational representation and reasoning is by inviting comparison and structural alignment. However, comparison‐based learning cannot often by itself account for children's ability to acquire relational concepts. Comparison is, by its nature, limited by *which* comparisons a child makes; and there is abundant evidence that children's early spontaneous comparisons are mostly limited to high‐similarity pairs (see Gentner & Hoyos, 2017, for a review). By itself, then, comparison would result in conservative learning—children would learn concepts whose members are highly similar to one another, such as basic‐level nominal concepts, but not perceptually variable concepts such as spatial relations. How do children overcome this obstacle? One proposal is that language invites comparisons that go beyond perceptual similarity (Christie & Gentner, 2010; Gentner, 2003; Yuan, Novack, Uttal, & Franconeri, 2024). That is, language interacts with comparison in important ways to facilitate relational learning.

### 1.2 Language can guide comparison to reveal relational insights

#### 1.2.1 Common labels invite comparison

Hearing the same label—even a novel label—applied to two different objects leads children to compare them in search of commonalities. For example, Gentner and Namy (1999) gave 4‐year‐olds a triad task in which everyday objects were given novel labels (e.g., a bicycle was labeled as “blicket”). When asked which of the two alternatives was also a blicket, children were equally likely to choose a perceptual shape match (a pair of glasses) or a conceptual taxonomic match (a skateboard). However, when they were shown *two* standards with the same labels before choosing (e.g., a bicycle and a tricycle were both labeled as “blicket”), they significantly favored the conceptual match. Critically, the results were not simply due to having two standards—when the two standards were labeled differently, children instead preferred the perceptual match (Namy & Gentner, 2002). These results suggest (1) that common labels can invite comparison, and (2) that the comparison process renders commonalities more salient, thereby promoting category learning (see also Hartley & Whiteley, 2024; LaTourrette & Waxman, 2019; Twomey, Ranson, & Horst, 2014; Waxman & Klibanoff, 2000). This is consistent with the long‐standing finding that labeling can promote categorical perception (Katz, 1963; Lupyan, 2008; Goldstone, Lippa, & Shiffrin, 2001).

#### 1.2.2 Naming promotes conceptual stability

A wide array of research has shown that having a label confers stability to the named concept and renders it more accessible for future use. As one example, consider the encoding of left‐right relations. Children under about 4–5 years typically do not spontaneously encode *left‐right* relations (Boone & Prescott, 1968; Dessalegn & Landau, 2008; 1994Hermer & Spelke, 1994, 1996; Hermer‐Vazquez, Moffet, & Munkholm, 2001; Miller, Vlach, & Simmering, 2017; Scott & Sera, 2018). One striking example comes from a study by Dessalegn and Landau (2008). Four‐ and five‐year‐olds were shown a two‐color square (e.g., red half left of blue half) and were immediately asked to pick the one they just saw from three alternatives. The results showed (1) that children were only 57% accurate at recognizing the correct target; and that (2) hearing relational language such as “Red is on the left” during encoding improved performance (79% correct). To understand the mechanism by which the labels affected performance, Yuan et al. (2024) utilized eye‐tracking in a similar paradigm and showed that a key benefit of relational language lies in guiding visual attention and encoding. Saying “Red is on the left of blue” helps 4‐year‐olds preferentially attend to and bind the featural information of color to its geometric information (e.g., left or right), thereby creating a more stable representation of the initial pattern. Importantly, children who received the relational language continued to show superior performance in further trials in which the language was no longer provided. These results suggest that language can foster relational encoding patterns that persist over time (Gentner, Özyürek, Gürcanli, & Goldin‐Meadow, 2013; Loewenstein & Gentner, 2005; Pyers & Senghas, 2009).

Spatial labels can act in concert to convey a larger spatial relational structure and guide systematic spatial encoding. For example, Loewenstein and Gentner (2005, Experiment 5) gave 3‐ and 4‐year‐old children a challenging mapping task in which the experimenter placed a toy at one of the three tiers of a “Hiding box” and asked children to find a hidden toy in the same place at the “Finding box.” Before starting the search trials, the Language group was asked to place a toy *at the top* (or *middle*, or *bottom*) in one box. For the Control group, the experimenter simply pointed to each location sequentially and asked the child to place a toy *here*. The hypothesis was that hearing interconnected spatial relational terms would invite children to form a structurally cohesive representation of the spatial structure of the box. This prediction was borne out: 3‐year‐olds in the *top‐middle‐bottom* group performed above chance in finding the analogous toy—significantly better than those in the control group, who performed at chance.^4^ The relational language advantage persisted over transfer to new structures; and, most importantly, it persisted over a 2‐day delay task in which only general language was used—evidence that the spatial terms invited children to form a stable relational representation. These findings suggest that language plays a role in shaping how relational concepts are encoded (Gentner, 2010, 2017; Gentner & Christie, 2010; Loewenstein & Gentner, 2005; Lupyan, Rahman, Boroditsky, & Clark, 2020; Mix, 2008; Scott & Sera, 2018; Shusterman, Ah Lee, & Spelke, 2011). Once learned, linguistic labels can help children retrieve relational representations to guide future encodings (Ankowski et al., 2012; Pruden, Levine, & Huttenlocher, 2011; Simms & Gentner, 2019; Yuan et al., 2024).

The above evidence suggests that language can act to further relational learning by inviting comparisons that children might not spontaneously make, and by promoting retention and reuse of named concepts. But our discussion so far is incomplete. We have focused only on studies in which language and comparison call attention to common structure. But comparison reveals differences as well as commonalities. We now turn to this phenomenon.

#### 1.2.3 Revealing alignable differences

The process of structural alignment highlights not only commonalities but also *alignable differences*—differences that play corresponding roles in the aligned structure (Gentner & Gunn, 2001; Markman & Gentner, 1993a, 1996; Sagi et al., 2012; Shao & Gentner, 2022). Evidence that alignable differences are specifically generated during the comparison process (rather than via joint processing or association) comes from a study by Gentner and Gunn (2001). Participants were given 20 pairs of concepts; half the participants were told to generate a commonality, and the other half to generate a thematic association between the concepts. (For example, the pair *tree‐child* could elicit the commonality “Both grow” or the thematic connection that “A child can climb a tree.”) Then, the participants were given the same 20 pairs, plus 20 new pairs (in random order), and were asked to write a *difference* between the items in the pair, for as many pairs as possible. Participants were told that they would not have time to finish them all, so they should pick the easy ones. If structural alignment is critical to noticing alignable differences, then carrying out a comparison task—even one focused on commonalities—should render alignable differences more available in the follow‐on task. The results bore out this prediction: the commonality group produced significantly more differences for the previously seen pairs than for the new pairs (the baseline), showing facilitation from the prior comparison task. Interestingly, the thematic group produced significantly *fewer* differences for the previously seen pairs than for the new (baseline) pairs.^5^

**Fig. 1:**
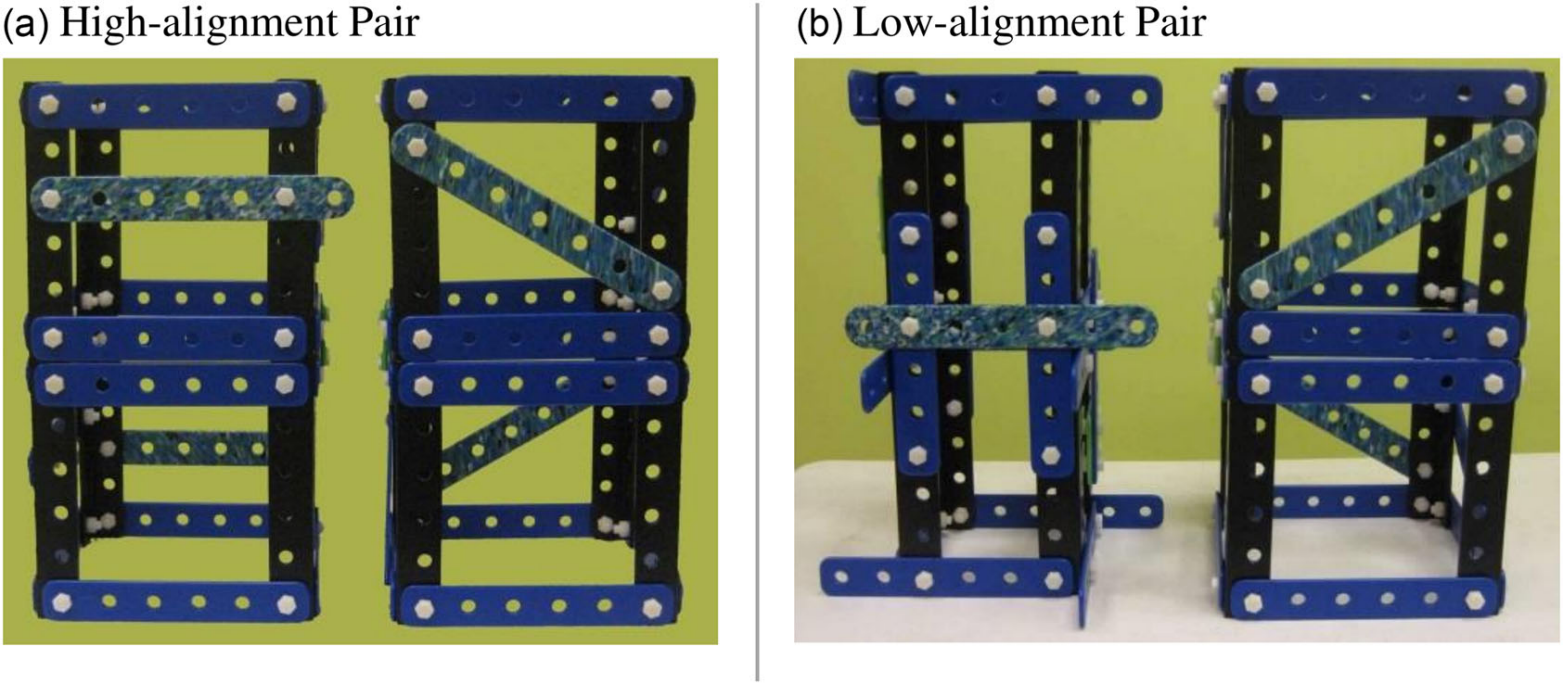
Model buildings used in the training. When the buildings are wiggled, the one with diagonal braces barely moves, while the one lacking diagonal braces leans almost to the ground.

The attention‐getting property of alignable differences offers a route by which children can come to notice nonobvious differences in spatial structure. For example, Gentner et al. (2016) sought to teach 6‐ to 8‐year‐olds the engineering principle that a diagonal brace confers stability—namely, that a triangle is a stable polygon whose shape cannot be changed without breakage, so the inclusion of triangles lends stability to a structure. The *diagonal* relation is an important spatial concept in engineering for children to master, as specified by the “Engineering is Elementary” curriculum and the International Technology and Engineering Educators Associations (ITEEA) standards of technological and engineering literacy. Yet, it is notably hard for children to acquire (Benjamin, Haden, & Wilkerson, 2010; Olson, 1970). In a study conducted in the Chicago Children's Museum, Gentner et al. (2016) tested whether comparison processes—specifically, close comparison that revealed an alignable difference—could aid children's learning. Children were shown two model buildings—one with a diagonal brace (which was, therefore, stable) and one with a horizontal crosspiece (which was unstable). In order to test the prediction that structural alignment was critical to success, there were two training groups: *high‐alignment* (HA) (in which the two buildings were highly surface‐similar) and *low‐alignment* (LA) (in which the buildings were surface‐dissimilar) (Fig. 1). All children wiggled the buildings, and all but a few (who were excluded from further analyses) found out that the braced one was more stable. A third group received no training but was simply given the two tasks of the test phase.

**Fig. 2:**
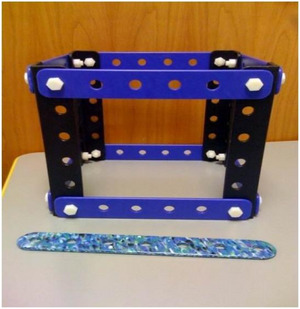
The one‐story building and beam used in the repair task.

Families first completed a construction task together, where they were asked to build a building as high as possible and were prompted to “brace your building so it won't fall.” Then, children were taken aside individually and given a brief “repair task.” They were shown an unstable model building (Fig. 2) and given a beam. The experimenter asked the child to show how to place the beam so as to make the building strong. The results showed, first, that this task was quite challenging—without training, even 8‐year‐olds were at chance in producing a diagonal brace. The results further showed a strong effect of alignment: children in the HA comparison were more likely to place the beam diagonally than those in the LA or control conditions, which did not exceed chance performance, even for 8‐year‐olds.

This shows that even a brief analogical comparison experience can help children gain insight into a critical but nonobvious difference—in this case, the use of a brace. Further, this insight was largely self‐driven; the experimenter did not describe or point out the brace. But the results also underscore the limits of spontaneous comparison in children. Even though all children recognized *which* building was stronger, only those in the HA condition reliably noticed *why* it was stronger. When the buildings were highly similar, children were likely to compare them and detect the key difference. But when the buildings differed in appearance, most children failed to achieve a structural alignment and thus failed to detect the alignable difference. This is consistent with prior findings that early in learning, spontaneous comparison is far more likely for closely similar cases than for dissimilar cases (Gentner, 2003; Gentner & Hoyos, 2017; Gentner & Rattermann, 1991; Gentner & Toupin, 1986; Loewenstein & Gentner, 2001; Paik & Mix, 2006).

In the present work, we ask whether *contrastive* relational language can act to promote the noticing of relational differences, even when perceptual alignability is low. That is, we ask whether contrastive labels can prompt contrastive comparison.^6^

### 1.3 The current study

In this paper, we ask whether contrastive language can invite contrastive comparison, and thereby foster children's discovery of a critical spatial concept. To do this, we extend Gentner et al.’s study on the use of comparison to promote children's understanding of the role of a diagonal brace in spatial construction, as just described. As in Gentner et al.’s study, we vary the alignability of the pairs children receive, enabling us to test whether HA will favor discovery of the brace principle, replicating the previous study. We break new ground by asking whether language—specifically, contrastive language—can foster comparison and thereby extend the power of comparison to LA pairs. Finally, an overarching goal of this research is to explore the extent to which children's own spontaneous comparison processes can drive their learning, without sustained pedagogical interaction. Therefore, we used fairly minimal instructions, as described below. For the same reason, we used the term *brace*,^7^ rather than a more familiar term such as *diagonal* or *criss‐cross*. (Pilot studies indicated that children at this age did not understand the meaning of *brace*.)

Experiment 1 had two phases. In phase 1, we replicated Gentner et al. (2016) method by examining the effect of the alignability of the training pairs on the elicitation of diagonal braces in the repair task. In phase 2, we introduced the language manipulation, crossed with the alignment factor. Half the children were in the label condition, and the other half were in the control condition. All children were again shown the two buildings and asked which one they remembered to be stronger (all answered correctly). In the control condition, the experimenter responded “Yes, this one is strong” and “that one is wobbly.” In the labeling condition, the experimenter responded “Yes, this one is strong. It has a brace” and that the wobbly building “does not have a brace.” Importantly, in order to mimic the effects of everyday incidental language, we did not point to the brace nor ask the child to identify it. Children then carried out a transfer task, in which they saw a new pair of buildings and had to decide which one was stronger. In Experiment 2, we both replicated and tested the robustness of learning by including new transfer tests and incorporating a delay.

Across the two studies, there are three main predictions. First, we predict that, as in Gentner et al. (2016) study, comparing a highly alignable pair should facilitate learning and transfer of the brace concept to a greater degree than comparing a dissimilar pair. Second, the use of a *brace* label should also facilitate learning and transferring the brace concept. Our third prediction, based on the prior findings reviewed above, is that the effects of language and alignment will persist over delay. Finally, we also explored a potential interaction between alignment and language. We asked whether the use of the *brace* label would diminish the HA advantage over LA, by inviting more comparison for LA pairs.

## 2 Experiment 1

### 2.1 Methods

#### 2.1.1 Participants

Sixty‐four 5‐ to 7‐year‐old children participated in the study (*M* = 6.4 years, *Range =* 5.5−7.3 years, 35 females). Assuming a power level of 80% and given the average observed effect size (odds ratio = 6.75) in Gentner et al. (2016), a total of 56 subjects was required. We had 64 because multiple families were recruited at the same time. Children were recruited through a database of families from a large Midwestern city in the United States. All had English as their primary language. Participants were evenly placed into the four training conditions so that each condition contained 16 children. No participants were excluded. The study took about 15 min. The families were compensated for participation.

#### 2.1.2 Design

The study had a 2×2 (Alignment × Language) between‐subjects design. The independent variables were Alignment (High [HA] vs. Low [LA]) and language (Label vs. Control). The dependent measures were the rate of brace responses in (1) the repair task and (2) the transfer task.

We had two key predictions in Experiment 1. First, we predicted an effect of alignment: that is, children in the HA condition would be more likely than those in the LA condition to register that the diagonal brace was important, and, therefore, to place a brace correctly (i.e., diagonally) in the Repair Task and to choose the braced building in the Transfer Task. Second, we predicted an effect of language: that is, children who heard the *brace* label would be more likely than those who did not in choosing the braced building in the Transfer Task. (We did not expect an effect of label on the Repair task performance because the Repair task was conducted before the language manipulation.) An open question was whether we would see an interaction between language and alignment in the Transfer Task.

#### 2.1.3 Materials and procedure

##### 2.1.3.1 Comparison training

After obtaining parental consent and the children's verbal assent, the experimenter brought the children into a quiet room and began the comparison training. Children were introduced to a pair of model buildings (Fig. 1; see also Gentner et al., 2016). One of the buildings had diagonal braces (and was, therefore, stable); the other had horizontal crosspieces instead (and was, therefore, unstable). The HA pair (Fig. 1a) consisted of two buildings that were identical in their overall structure except for the critical difference that one building had two diagonal braces, and the other building had horizontal crosspieces in the corresponding locations. The LA pair (Fig. 1b) also consisted of a braced building and an unbraced building, but the two buildings were quite dissimilar in appearance. While the braced building was the same as in the HA pair, the other building was narrower, with beams protruding from the top and bottom and horizontal crosspieces located in the middle. The prediction was that the LA pair would be more difficult to align than the HA pair.

Half the children saw the HA pair, and the other half saw the LA pair. Whether the braced building was on the left or right was randomized. The experimenter first asked, “*Which building do you think is stronger*”.^8^ Seventy‐six percent of children answered correctly, and there was no group difference. The experimenter then invited the children to wiggle both buildings to find out which one was truly stable. If children declined to wiggle, the experimenter did it for them. After wiggling, the experimenter asked again which building was stronger. All children were then able to answer correctly that the braced building was stronger. However, the key question remained as to whether children would notice *why* the braced one was stronger.

##### 2.1.3.2 Repair task

After a 3‐min filler task, the experimenter brought out a wobbly one‐story building frame lacking interior pieces (Fig. 2). He wiggled it to demonstrate that it was unstable and commented, “*Someone made this building*, *but it still wobbles*.” Then, he handed a beam to the children and asked, “*Can you help me make it stronger? Can you make it so that it doesn't wobble?*” (We did not require that the child screw in the beam; it was enough for the child to hold the beam up to the building.) The first dependent measure was children's placement. Either diagonal placement was scored as 1 (correct); a horizontal or vertical placement was scored as 0 (incorrect).

##### 2.1.3.3 Reminding and language

The language phase immediately followed the repair task. Half the children from each group (16 in HA and 16 in LA) were assigned to the Label group; the other half were the Control group. The experimenter took out the same model buildings used in the comparison training and asked children “Do you remember which building is strong?” All children were able to point to the correct building. For those in the Label condition, the experimenter said, “*Yes*, *this building* (waving to the braced building) *is strong. It has a brace*, *but this one* (waving to the other building) *is wobbly. It does not have a brace*.” In saying the sentence, the experimenter took care not to point to any particular part of the buildings but only waved at each building from a distance. For the Control group, the experimenter said “*Yes*, *this building* (waving to the braced building) *is strong*, *but this one* (waving to the other building) *is wobbly*.”

##### 2.1.3.4 Transfer task

**Fig. 3:**
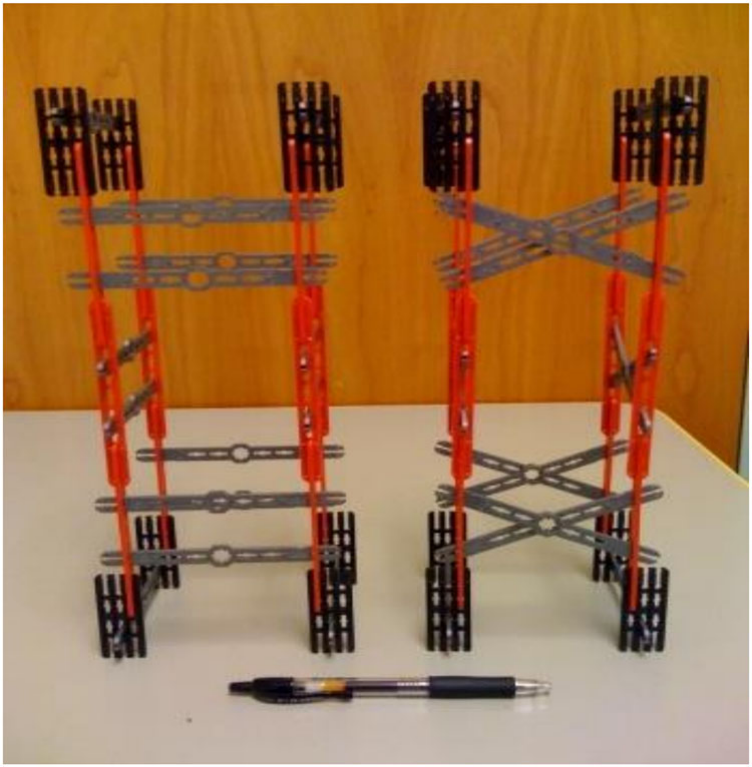
The pair of tall buildings used in the transfer task.

After another 3‐min filler task, the experimenter took out a pair of tall buildings for the transfer task (Fig. 3). As with the training buildings, one of the tall buildings contained two diagonal pieces forming braces and was, therefore, stable; the other building had horizontal crosspieces instead and was unstable. Children were told, “*One of these buildings is strong and one of them is wobbly. Which one do you think is strong?*” The second dependent measure was children's choice: choosing the braced building was scored as 1, and choosing the one with all horizontal crosspieces was scored as 0.

### 2.2 Results

**Fig. 4:**
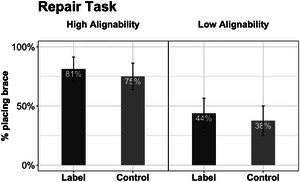
Results of Experiment 1: Repair task performance by conditions. The results for Label and Control groups are shown for comparison with further results, although the label was not used until later. Error bars represent standard errors.

**Fig. 5:**
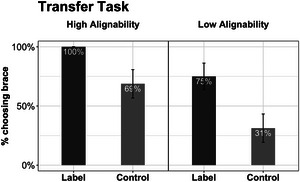
Results of Experiment 1: Transfer task performance by conditions. Error bars represent standard errors.

To preview, the results bear out our main hypotheses regarding the benefits of alignment and relational labels. Children in the HA comparison condition were more likely to learn the brace principle than those in the LA condition, and children who heard the *brace* label were more likely to learn the principle than those who did not. All analyses were conducted in R (Version 4.2.3; R Core Team, 2024). Since both tasks were coded binarily (with placing or choosing a brace as 1 and others as 0), we conducted logistic regressions for each, with alignment (HA vs. LA) and language (Label vs. Control) as the independent variables. The results of the logistic regressions are summarized in Table 1, 9 and Figs. 4 and 5. Mean performance reported in text was calculated by averaging participants’ responses with respect to the relevant conditions.

**Table 1:** Experiment 1: Summary of main results

Task	Factor	*b*	*p*‐value	Effect size	95% CI
Repair	Intercept	0.449	.11	1.57	[0.91, 2.77]
	Alignment	1.661	.003	2.31	[1.35, 4.14]
	Language	0.304	.58	1.17	[0.68, 2.03]
Transfer	Intercept	1.139	.002	3.12	[1.63, 6.78]
	Alignment	1.938	.005	2.66	[1.39, 5.59]
	Language	2.278	.001	3.15	[1.63, 6.88]

*Note*. Variables are centered for this analysis.

Preliminary logistic regressions were conducted for whether age and gender predicted children's performance. Results showed that age did not significantly predict performance in either task (*p*s > .81). Gender marginally predicted Repair performance (*M*_boys_ = .72; *M*_girls_ = .49), *p* = .056, and significantly predicted Transfer performance (*M*_boys_ = .83; *M*_girls_ = .57), *p* = .034. In both tasks, boys outperformed girls. However, since gender did not interact with either alignment or language^10^ (*p*s > .54), data were collapsed across both age and gender.

#### 2.2.1 Repair task

The logistic model for the repair task revealed a significant effect of alignment (Fig. 4). Children who saw the HA pair were more likely to place a diagonal brace in the repair task than those who saw the LA pair (*M_HA_* = 0.78, *SD_HA_* = 0.42; *M_LA_* = 0.41, *SD_LA_* = 0.50). There was no effect of language, as the label manipulation occurred only after the repair task (*M_Label_* = 0.62, *SD_Label_* = 0.49; *M_Control_* = 0.56, *SD_Control_* = 0.50). Thus, in the absence of linguistic cues, the difference between HA and LA conditions was fairly dramatic—as in Gentner et al. (2016) study, only the HA group exceeded chance.

#### 2.2.2 Transfer task

The logistic model for the transfer task revealed significant effects of both alignment and language (Fig. 5). Children who saw the HA buildings were more likely to choose the braced building as the stronger one than those who saw the LA buildings (*M_HA_* = 0.84, *SD_HA_* = 0.37; *M_LA_* = 0.53, *SD_LA_* = 0.53). And those who heard the *brace* label were more likely to choose the braced building than those who did not (*M_Label_* = 0.88, *SD_Label_* = 0.34; *M_Control_* = 0.50, *SD_Control_* = 0.51).

Together, the results confirmed our first hypothesis: seeing highly alignable buildings helped children compare and notice the alignable difference between the diagonal beam and the horizontal beam—a key prerequisite for learning the brace principle. The results also supported our second hypothesis: children who heard the *brace* label were more likely to learn and transfer the idea of bracing.

We next explored the interaction between alignment and language. This was done to address the question of whether language was especially beneficial in the LA condition. Prior research (and our results so far) indicates that LA is less likely to lead to spontaneous comparison than HA. If applying the label invites comparison, it might act to “level the playing field,” reducing the difference between HA and LA. To test this possibility, we subtracted the number of LA children who passed the Transfer task from that of the HA group within each language condition. This difference in frequency indicates the magnitude of the advantage of HA over LA. A Chi‐square test of independence failed to show a significant difference between the label and no‐label conditions, *p* = .70. For converging evidence, we also reran the regression model, including the interaction term, and conducted a nonparametric bootstrapping analysis (see Supplementary Materials). All analyses failed to show a significant interaction. Thus, we failed to find an interaction between alignment and language.

### 2.3 Discussion

The results from Experiment 1 replicate and extend prior findings concerning the benefits of structural alignment. Our first hypothesis was that HA would facilitate difference‐detection. Consistent with Gentner et al. (2016) findings, children who received the highly alignable training pair were more likely to use a diagonal brace in repairing a building frame than those who received the LA pair. The results also bore out our second hypothesis, that contrastive labels can facilitate difference‐detection. Children who briefly heard the *brace* label applied to the stable building were more likely to identify the stronger transfer building than those who did not receive such a label. We did not find evidence for an interaction between alignment and language in learning the brace principle. Language facilitated learning in both HA‐ and LA training conditions.

In Experiment 2, we tested the same set of hypotheses as well as Prediction 3—that the effects of language and of alignment would persist over delay. To do so, we brought children back after a 2‐ to 5‐day delay and included a greater variety of transfer tasks. Testing transfer—especially after a delay—is critical to our thesis that these processes help children build long‐term cognitive skills. Indeed, transfer is often considered the “true test of learning” and is a primary goal of formal science education (see Barnett & Ceci, 2002; Klahr & Chen, 2011). On Day 1, we administered the training and reminding phases and the repair task similar to Experiment 1. On Day 2, children again received the repair task; in addition, they received the near‐transfer task with two novel buildings (as in Experiment 1), and two far‐transfer tasks on unstable bunkbeds. We reasoned that if alignment and language highlight the difference of diagonal brace through comparisons, children should be more likely to apply this knowledge to novel situations, even dissimilar ones such as the bunkbed. In addition, if relational labels help reify relational concepts, children in the Label condition should be able to retain their learning over delay (Loewenstein & Gentner, 2005; Yuan et al., 2024).

## 3 Experiment 2

### 3.1 Method

#### 3.1.1 Participants

The participants were 136 5‐ to 7‐year‐old children (*M* = 6.1 years, *Range =* 5.0−7.4 years, 60 females). Fifteen participants were excluded for not returning for the Day 2 session (due to COVID‐related reasons). Another five participants were excluded for failing the memory check (see below), leaving a final sample size of 116. Participants were evenly placed into the four training conditions (2 Alignment × 2 Language) such that each condition contained 29 children. The sample size was planned based on the power analysis for the interaction effect between alignment and language, using the R package “interactionPoweR” (Baranger et al., 2023). Based on the observed phi coefficient between alignment, language, and transfer performance in Experiment 1, a sample size of 29 was required to detect the interaction with 80% power. Children were recruited through a database of families from a large Midwestern city in the United States. All had English as their primary language. Each session took around 15 min.

#### 3.1.2 Design

The study had a 2×2 (Alignment × Language) between‐subjects design and five dependent variables (Day 1: Repair; Day 2: Repair, Near Transfer, Far Transfer [Construction], and Far Transfer [Recognition]). The independent variables were the alignment manipulation (HA vs. LA) and the language manipulation (Label vs. Control). The dependent measures were the rate of brace responses in each task.

There were three predictions. First, as in Experiment 1, we predicted that children in the HA condition would be more likely than those in the LA condition to notice the critical difference of diagonal brace versus horizontal piece and to transfer this insight to other tasks. Second, we predicted that children who heard the *brace* label would be more likely than those who received the control language to learn and transfer the idea of bracing. The third prediction concerns robustness over delay: we predicted that both language and alignment would facilitate retaining the brace idea over a 2‐ to 5‐day delay. Finally, we explored the interaction between alignment and language.

#### 3.1.3 Materials and procedure

##### 3.1.3.1 Day 1 training

Half the children (58 out of 116) were assigned to the HA comparison condition (HA; Fig. 1a) and the other half to the LA comparison condition (LA; Fig. 1b). The comparison training procedure was the same as in Experiment 1. The experimenter asked children to guess which of the two model buildings was stronger; 63% of children answered correctly, and there was no group difference. The experimenter then invited them to check by wiggling the buildings. All children reported correctly that the braced one was stronger. The language manipulation followed immediately. In the Label condition, the experimenter confirmed the children's observation by saying, “*Yes*, *this building* (waving at the braced building) *is strong. It has a brace*, *but this one* (waving at the unbraced building) *is wobbly. It does not have a brace*.” The Control condition simply heard, “Yes, *this building* (waving at the braced building) *is strong*, *but this one* (waving at the unbraced building) *is wobbly*.” Half the children in each comparison condition (29 out of 58) received the *brace* label, and the other half received the control language.

##### 3.1.3.2 Day 1 repair

After a 3‐min filler task, the experimenter brought out a wobbly one‐story building and asked children to make it stronger (Fig. 2). The procedure of the repair task was the same as in Experiment 1—children were handed a beam and asked how they could make the building stronger (as before, it was enough for the child to hold the beam up to the building). The first dependent measure was the placement of the beam. Placing the beam diagonally was scored as 1, and placing it vertically or horizontally was scored as 0.

##### 3.1.3.3 Day 1 training reminder

After another short filler task, the experimenter brought out the initial training buildings and asked children whether they remembered which one was stronger. (All except one child correctly identified the stronger building. For this child, the experimenter wiggled the buildings again and reminded them that the braced one was stronger.) Then, the experimenter repeated the same language manipulation as before: “*Yes*, *this building is strong. It has a brace…*” (Label) or “*Yes*, *this building is strong*, *but that one is wobbly*.” (Control).

##### 3.1.3.4 Day 2 repair

After a 2‐ to 5‐day delay, children returned to the lab. They first completed the same repair task as on Day 1. The second dependent measure was children's score on this task—1 for either diagonal placement, and 0 for a horizontal or vertical placement.

##### 3.1.3.5 Day 2 near transfer

The near‐transfer task was the same as the transfer task in Experiment 1. The experimenter brought out the two tall buildings (Fig. 3) and asked children to guess which one was stronger. Thus, the third dependent measure was children's choice (1 for choosing the braced building, 0 for choosing the unbraced building).

##### 3.1.3.6 Day 2 far transfer tasks

**Fig. 6:**
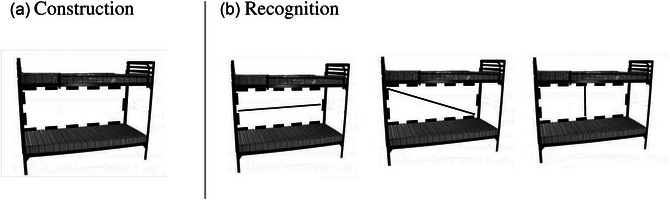
The bunkbed pictures used in the far transfer task.

The far transfer tasks consisted of two parts: construction and recognition. For the construction task, the experimenter showed children a picture of a bunkbed (Fig. 6a) and said, “*Right now the bed wobbles when someone wants to sleep in it. The bunk bed is missing a piece that will keep it from wobbling. Draw a single line to keep the bed from wobbling*” (adapted from Applebaum, 2014). If children showed hesitance or drew something irrelevant (e.g., a ladder or decorations), the experimenter further prompted them that they needed to draw a line within the bed frame. If children drew multiple lines, the experimenter asked which line made the bed strongest and based the scoring on the final answer. Thus, the fourth dependent measure was whether children drew a diagonal brace (scored as 1) or something else (scored as 0). This construction task was expected to be challenging.

For the recognition task, the experimenter laid down three depictions of the same bunkbed side by side, each with a line added to the frame (horizontally, vertically, and diagonally; Fig. 6b). Children were asked to choose which bed was now the strongest. The experimenter said, “*Three of my friends drew a line to show how a piece could make it stronger. Alex drew this one*, *Freddy drew this one*, *and Sally drew this one. Which one do you think is stronger*, *Alex's*, *Freddy's*, *or Sally's?*” The order of the drawings was randomized. The fifth dependent measure was the children's choice of drawing (1 for choosing the bed with the diagonal brace, 0 for choosing either of the other two options).

##### 3.1.3.7 Day 2 memory check

At the end of the study, children were given a memory check. They were again shown the two initial training buildings and were asked, “Can you remember which one is stronger? Can you point to it for me?” Children who failed this memory question were excluded from the study (*N* = 5; one in the HA & Label condition and four in the HA & Control condition). There were three follow‐up questions to explore the strength of children's learning. After pointing, children were asked, “*Why do you think it's strong?*” and “*Can you point to what makes that one strong?*” The Label group received one additional question. The experimenter pointed to the diagonal piece in the braced building and asked, “*What do we call this?*” The results of the three follow‐up questions are reported in the Supplementary Materials.

### 3.2 Results

**Fig. 7:**
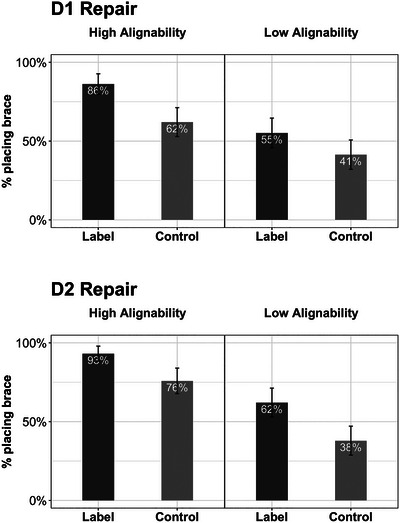
Results of Experiment 2: Repair task performance by conditions. The top and bottom panels show Day 1 and Day 2 performance, respectively. Error bars represent standard errors.

**Fig. 8:**
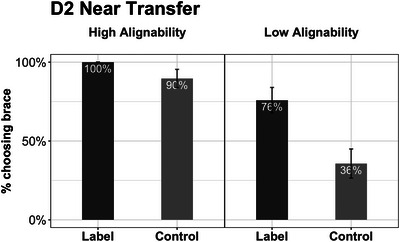
Results of Experiment 2: Near transfer task performance by conditions. Error bars represent standard errors.

**Fig. 9:**
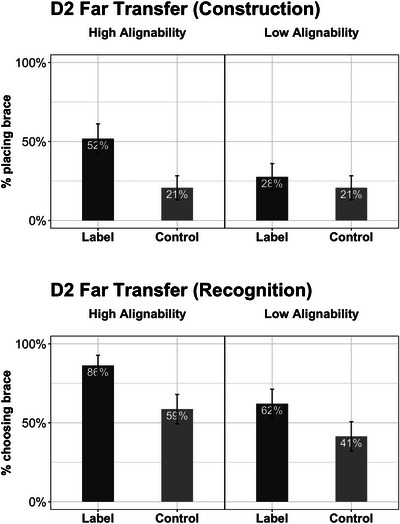
Results of Experiment 2: Far transfer task performance by conditions. The top and bottom panels show the construction and the recognition performance, respectively. Error bars represent standard errors.

The results again bore out our main hypotheses: there were positive effects of both HA and contrastive label. Logistic regressions were conducted with alignment (HA vs. LA) and language (Label vs. Control) as the independent variables and each of the five measures as the dependent variable. The results are summarized in Table 2 and Figs. 7, 8, 9.

**Table 2:** Experiment 2: Summary of main results

	Task	Factor	*b*	*p*‐value	Effect size	95% CI
Day 1	Repair	Intercept	0.514	.023	1.67	[1.13, 2.52]
		Alignment	1.172	.004	1.80	[1.21, 2.73]
		Language	0.877	.032	1.55	[1.05, 2.34]
Day 2	Repair	Intercept	0.902	.000	2.46	[1.59, 3.99]
		Alignment	1.803	.000	2.47	[1.59, 4.02]
		Language	1.128	.012	1.76	[1.14, 2.79]
	Near Transfer	Intercept	1.774	.000	5.89	[3.16, 13.07]
		Alignment	2.976	.000	4.46	[2.41, 9.75]
		Language	1.877	.001	2.57	[1.51, 4.62]
	Far Transfer (Construction)	Intercept	−0.900	.009	0.41	[0.26, 0.61]
		Alignment	0.605	.15	1.36	[0.90, 2.07]
		Language	0.941	.027	1.60	[1.06, 2.47]
	Far Transfer (Recognition)	Intercept	.555	.007	1.74	[1.17, 2.64]
		Alignment	0.960	.019	1.62	[1.09, 2.45]
		Language	1.109	.007	1.75	[1.17, 2.65]

*Note*. Variables are centered for this analysis.

Preliminary logistic regressions on age and gender showed that age did not significantly predict performance in any tasks (*p*s > .07). Gender only significantly predicted the Day 2 bunkbed construction task (*M*_boys_ = .42; *M*_girls_ = .18), *p =* .005, wherein boys were more likely than girls to draw a brace on the bunk bed picture. Since gender did not interact with alignment or language (*p*s > .34), data were collapsed across age and gender. In addition, the number of days between Days 1 and 2 did not predict performance on any measure, *p*s > .43.

#### 3.2.1 Day 1 repair task

The logistic regression for the Day 1 repair task revealed significant effects of both alignment and language (Fig. 7, top panel). Children who saw the HA buildings were more likely to place the beam diagonally in the repair task than those who saw the LA buildings (*M_HA_* = 0.74, *SD_HA_* = 0.44; *M_LA_* = 0.48, *SD_LA_* = 0.50). Those who heard the *brace* label were more likely to place the beam diagonally than those who heard the control language (*M_Label_* = 0.71, *SD_Label_* = 0.46; *M_Control_* = 0.52, *SD_Control_* = 0.50).

#### 3.2.2 Day 2 repair task

The remaining tasks were all given on Day 2 (Fig. 7, bottom panel). The logistic regression for the Day 2 repair task also revealed effects of alignment and language. Children who saw the HA buildings were more likely to place a brace than those who saw the LA buildings (*M_HA_* = 0.84, *SD_HA_* = 0.37; *M_LA_* = 0.50, *SD_LA_* = 0.50). Likewise, children who heard the *brace* label were more likely to do so than those who heard the control language (*M_Label_* = 0.78, *SD_Label_* = 0.42; *M_Control_* = 0.57, *SD_Control_* = 0.50).

In addition, we compared the results of the two repair tasks to check whether Day 2 might show a performance drop due to forgetting. Interestingly, performance did not differ between Day 1 and Day 2 (*M_D1_* = 0.61, *SD_D1_* = 0.49; *M_D2_* = 0.67, *SD_D2_* = 0.47), *p =* .41—evidence that learning was robust.

#### 3.2.3 Day 2 near transfer

The logistic regression for the near transfer task revealed significant effects of alignment and language (Fig. 8). As predicted, the HA group was more likely than the LA group to choose the braced building as the stronger one (*M_HA_* = 0.95, *SD_HA_* = 0.23; *M_LA_* = 0.56, *SD_LA_* = 0.50). Also, as predicted, the Label group was more likely to choose the braced building than was the Control group (*M_Label_* = 0.88, *SD_Label_* = 0.33; *M_Control_* = 0.63, *SD_Control_* = 0.49).

#### 3.2.4 Day 2 far transfer (construction)

As expected, the overall performance on the far transfer construction task was low (Fig. 9, top panel). The logistic regression revealed a significant effect of language: the Label group was more likely to draw a brace than was the Control group (*M_Label_* = 0.40, *SD_Label_* = 0.49; *M_Control_* = 0.21, *SD_Control_* = 0.41). However, there was no effect of alignment. The HA and LA groups performed similarly (*M_HA_* = 0.36, *SD_HA_* = 0.48; *M_LA_* = 0.24, *SD_LA_* = 0.43) in this construction task.

#### 3.2.5 Day 2 far transfer (recognition)

Not surprisingly, performance on the recognition task was better than that on the construction task (Fig. 9, bottom panel). The logistic regression revealed significant effects of both alignment and language. The HA group was more likely to choose the braced bed as the strongest one than the LA group (*M_HA_* = 0.72, *SD_HA_* = 0.45, *M_LA_* = 0.52, *SD_LA_* = 0.50). And the Label group was more likely to choose the braced bed than was the Control group (*M_Label_* = 0.74, *SD_Label_* = 0.44, *M_Control_* = 0.54, *SD_Control_* = 0.50).

Overall, the results confirm all three of our predictions. On Day 1, when tested soon after training, first, children in the HA condition outperformed children in the LA condition; and, second, children who received the *brace* label outperformed those who received neutral control language. Our third prediction was that the effects of both alignment and contrastive labeling would be robust across a delay. Consistent with this prediction, both factors continued to show a statistically significant advantage on all but one of the four Day 2 tasks (Table 2). On the construction version of the Far Transfer task, alignability had no significant effect. This was by far the most difficult task; interestingly, labeling remained significant on this task.

Last, we explored the interaction of alignment and language by checking whether the difference in performance between HA‐ and LA was greater for the Control condition than for the Label condition. Again, we subtracted the number of LA children who passed the tasks from that of HA children and ran Chi‐square tests on this difference in frequency by the language conditions (see Supplementary Materials for additional analyses). Table 3 summarizes the results. Out of the five tasks, labeling only significantly diminished the difference between LA‐ and HA for the Near Transfer task (for which there appears to be a ceiling effect in the HA condition). Further, contrary to our prediction, labeling significantly increased this difference for the Far Transfer construction task. Labeling had no effect in the two Repair tasks, nor in the Far Transfer recognition task. Overall, there was no consistent pattern of interaction between alignment and language.

**Table 3:** Experiment 2: Summary of interaction results

	Task	Condition	Difference in frequency	*p*‐value of Chi‐square	Pattern
Day 1	Repair	Label	9	.6	No interaction
		Control	6		
Day 2	Repair	Label	9	.8	No interaction
		Control	11		
	Near Transfer	Label	6	.03	In accord with the expected direction
		Control	16		
	Far Transfer (Construction)	Label	7	.02	Opposite to the expected direction
		Control	0		
	Far Transfer (Recognition)	Label	7	.7	No interaction
		Control	5		

## 4 General discussion

In two experiments, we found evidence that contrastive language and comparison can help 5‐ to 7‐year‐old children learn a critical but nonobvious spatial concept—the use of a diagonal brace to confer stability to constructions. There are three main findings. First, we replicated prior findings (Gentner et al., 2016) showing that comparing two highly alignable model buildings fostered the discovery of a critical spatial concept—the brace. Second, we found that contrastive language supported contrastive comparison—that is, children who heard a term (*brace*) applied contrastively were more likely to notice and use a diagonal brace in subsequent tasks. Third, we found that both the effect of alignment and the effect of contrastive labels were robust over transfer and delay.

### 4.1 The effect of alignment

The two experiments provide evidence that comparison can highlight nonobvious differences in spatial structures and that this can enable learners to notice and retain those differences. In Experiment 1, children viewed pairs of model buildings that were either highly similar overall (HA group) or surface‐dissimilar (LA group). In both cases, one building had a diagonal bar (a brace) and the other instead had a horizontal crosspiece. All children agreed that the building with the diagonal brace was strong and the other was wobbly. Yet, in a later “repair” task, roughly twice as many children in the HA condition used a diagonal placement to stabilize a building—evidence that structural alignment is important in generating this insight.

In Experiment 2, we replicated the effects of alignment and contrastive labels as found in Experiment 1. We then went on to test the robustness of learning by retesting after a 2‐ to 5‐day delay and by including both near and far transfer tasks. Children in the HA condition continued to perform well after the delay. In all measures (except for the drawing task, on which children mostly failed), we found a significant advantage of HA training over LA training in children's use and recognition of diagonal bracing. The finding that high surface similarity can facilitate comparison and difference detection is consistent with the results of Gentner et al. (2016) study in the Chicago Children's Museum. More broadly, our results dovetail with those of many other studies in finding that HA comparison facilitates noticing alignable differences (Gelman, Raman, & Gentner, 2009; Gentner & Gunn, 2001; Matlen, Gentner, & Franconeri, 2020; Sagi et al., 2012; Zheng, Matlen, & Gentner, 2022).

### 4.2 The effect of language

Our second prediction—that hearing a contrastive label would promote contrastive comparison—was also supported. In both studies, children who received the contrastive label *brace* were more likely to learn and retain the idea of a diagonal brace than those who did not. We suggest that the contrastive language (“This one …*has a brace*” vs. “This one…*does not have a brace*”) signaled that there was a relevant difference between the buildings. Thus, children who had not already noticed the brace were invited to re‐examine the buildings and compare (or recompare) them, paying special attention to any differences that emerged from the mapping process—in this case, the diagonal bracing present only in the stable building.^11^ For children who had already noticed the diagonal relation, the contrastive term *brace* may have linked with that relation, increasing the likelihood that the relation would be retained for future use.

### 4.3 Contrastive language and contrastive comparison

The finding that comparison and structural alignment can aid learning is not new. Much prior work has shown that comparison can reveal common structure (e.g., Catrambone & Holyoak, 1989; Doumas & Hummel, 2013; Gelman et al., 2009; Gick & Holyoak, 1983; Kurtz, Miao, & Gentner, 2001; Loewenstein, Thompson, & Gentner, 1999; Murphy, Zheng, Shivaram, Vollman, & Richland, 2021), and that common labels invite comparison (Chen, Mo, & Honomichl, 2004; Christie & Gentner, 2010; Gelman & Markman, 1985; Gentner et al., 2011; Gentner & Namy, 1999; Waxman & Klibanoff, 2000). Likewise, the finding that comparison can invite noticing alignable differences is also well‐supported (Gelman et al., 2009; Gentner & Gunn, 2001; Markman & Gentner, 1993a; Markman & Gentner, 1996; Sagi et al., 2012). However, comparatively little work has examined the effects of language in inviting contrastive comparison. Our findings show that contrastive labels can invite comparison and thereby potentiate noticing alignable differences.

### 4.4 Implicit comparison and everyday learning

One goal of this work was to explore the idea that children learn from comparisons that are implicit in adults’ use of language, and not necessarily intended as pedagogy. This is important to our overarching interest in exploring learning as the key driver of cognitive development. Therefore, we used a minimal comparison prompt: “Which building is stronger?” This contrasts with the methods used in prior studies of comparison‐based learning, which have given children explicit prompts to compare. For example, in Loewenstein and Gentner's (2001) study, described earlier, children were asked “Do you see how these [the two model rooms] are alike?” Similar prompts were used by Gentner and Namy (1999) and Christie and Gentner (2010). In other cases, explicit instructions to find item correspondences have been used to facilitate comparison (DeLoache, 1989; Richland & McDonough, 2010). In the present studies, the experimenter never explained or pointed to the brace. Also, beyond answering which building was stronger, children were not asked to think about the commonalities and differences between the braced and unbraced buildings. Yet, in both experiments, children who heard the *brace* label showed better understanding of the brace concept and retained this understanding over a few days’ delay. This suggests that even fairly casual exposure to relational language may allow children to gain a new relational concept.

Our findings can be compared to work in language acquisition that has used implicit comparison to support children's acquisition of word meaning. For example, in a classic study by Carey and Bartlett (1978), young children were shown two trays, identical except for color, and asked to “Bring me the *chromium* one, not the *red* one.” Although the task only required children to select the not‐red tray, many of the children learned the novel color term (*chromium*). In order to test whether implicit comparison between the two trays was important in children's success in this task, Shao and Gentner (2022, Experiment 1) repeated the study, varying the initial materials. Half the children saw two objects that were highly alignable, as in Carey and Bartlett's study (e.g., a chromium circle and a blue circle); the other half saw a less alignable pair (e.g., a chromium circle and a blue square). Three‐ and four‐year‐old children were asked to “point to the chromium one, not the blue one.” Both groups were highly accurate (96.9%) in choosing the correct (chromium) object during the initial task; but only those who received the high‐alignable pair learned the meaning of *chromium* (as evidenced by later passing a yes‐no “Is this one chromium?” task). Children in the LA group, despite being nearly perfect at choosing the “not‐blue” object, performed at chance (3‐year‐olds) or just above chance (4‐year‐olds) on the yes‐no chromium recognition test. Shao and Gentner (2022) suggested that viewing the highly alignable pair prompted an alignment process that led to the popout of the alignable difference in color. Children then spontaneously connected that salient color to the *chromium* label. In our study, as in Shao and Gentner's study, the use of contrasting language also prompted a comparison that led children to notice specific differences. We suggest that children's ability to learn from incidental language and implicit comparison may help explain their rapid gains in knowledge.

### 4.5 Long‐term effects of language

In our study, the *brace* label, once linked to a difference, increased the likelihood of retaining the difference in a delayed task. This finding joins other evidence that language can reify the named concept, making it more stable in memory (Gentner & Christie, 2010; Pyers & Senghas, 2009; Pyers, Shusterman, Senghas, Spelke, & Emmorey, 2010; Scott & Sera, 2018; Shao & Gentner, 2022; Yuan et al., 2024). One striking example comes from a longitudinal study by Pruden et al. (2011), in which they recorded the spatial language of 52 parent−child dyads from 14 to 46 months of age. They found that the number of different spatial terms a child produced between 14 and 46 months predicted their scores on three nonverbal tests of spatial ability at 54 months. As the authors concluded, “An enhanced ability to verbally describe spatial features and spatial relations may lead to an increased awareness and better encoding of spatial features and relations (Gentner, 1988; Gentner, Simms & Flusberg, 2009)” (Pruden et al., 2011, p. 1428). As further support, Loewenstein and Gentner (2005) found that 3‐year‐old children who heard the terms *top‐middle‐bottom* in a spatial mapping task were able to use this connected relational structure to transfer knowledge to new structures without reinstating the language, even after a 2‐day delay. The present study goes further in showing that even a novel term can reify a relational concept. The 5‐ to 7‐year‐old children in our studies did not initially know the meaning of *brace*, yet those who received the label still showed improved insight into the corresponding concept, both immediately and over delay.

These findings are consistent with the proposal that language and other conventional symbols help create our cognitive toolkit (Gentner, 2010, 2016; Gentner & Christie, 2010; Gentner & Hoyos, 2017). According to this proposal, the habitual use of the same label across situations renders its meaning more stable and more accessible in memory. This encoding may come to be sufficiently entrenched as to become spontaneously evoked in novel situations, whether or not the word is used (Forbus, Gentner, & Law, 1995; Gentner et al., 2013; Jamrozik & Gentner, 2020; Miller & Simmering, 2018). Evidence comes from a study comparing hearing 6‐year‐old Turkish homesigners^12^ with Turkish hearing children (Gentner et al., 2013). By 6 years of age, Turkish hearing children typically have a full vocabulary of spatial relations, including *top*, *middle*, *bottom*. In contrast, the homesigners, despite having invented a rich gestural language for objects and events, appeared to have a greatly impoverished spatial lexicon.^13^ To explore whether this lack of spatial symbols affected the homesigners’ ability to encode and reason about spatial relations, the researchers compared the homesigners with matched Turkish hearing children on a spatial mapping task. As in Loewenstein and Gentner's (2005) task, the experimenter placed a toy in a three‐tiered Hiding box and asked the child to find the analogous toy in the same place at the Finding box. No spatial terms were provided to either group, so children had to rely on their own encoding. The results were fairly dramatic: the hearing children performed quite well, but the homesigners performed at chance. The authors suggested that the hearing children, with their well‐entrenched spatial language, naturally encoded the two boxes in the same way, facilitating their ability to map between them. The homesigners, lacking this resource, did not succeed. These findings provide evidence that language can confer long‐term benefits in spontaneous relational encoding. (But see Karadöller et al., 2022 for negative findings on language effects.^14^)

We suggest that stable relational encodings also favor uniform encoding of relations across contexts, facilitating comparison and transfer (Forbus et al., 2005; Gentner et al., 2013). For example, when given an ambiguous sorting task, college students who majored in an integrated STEM curriculum were highly likely to sort phenomena into groups that shared causal structure (e.g., *positive feedback*, *common cause*, etc.); in contrast, novices in physical science (psychology and economics majors) mostly sorted by domain (electronics, ecology, etc.) (Rottman, Goldwater, & Gentner, 2012). We suggest that through habitually labeling causal patterns, and comparing cases in terms of their causal structure, experts form long‐term representations in which key causal patterns are prominently represented. This may explain the finding that experts are better able to retrieve relationally similar phenomena than are novices (Novick, 1988; see also Goldwater et al., 2021; Goldwater & Jamrozik, 2019; Jamrozik & Gentner, 2020). In short, we suggest that acquiring relational language is a major contributor to conceptual development.

### 4.6 Limitations and clarifications

Although we have emphasized the role of language in cognition, we are not arguing that language is the only route to learning useful habitual encodings. For example, research in perceptual learning has shown that perceptual systems can undergo long‐lasting changes in response to practice and environmental demands (e.g., Goldstone, 1998). One mechanism is through attentional weighting, where attention is preferentially allocated to important perceptual features and dimensions. For example, Goldstone (1994) showed that training on simple categorization rules (e.g., by brightness) sensitized people's later perceptual discrimination of the category‐relevant dimension (e.g., brightness), while in some cases desensitizing that of an irrelevant dimension (e.g., size). Another phenomenon of perceptual learning is that unfamiliar parts that are important in one task are more likely to be used in representing subsequent categories (Schyns & Rodet, 1997). As Goldstone (1998, p. 592) notes, this kind of perceptual learning can lead to “the development of new ‘building blocks’ for describing stimuli.”

A second point that may need discussion is our usage of the term *relational category*. We follow the definition offered by Gentner and Kurtz (2006): “By *relational category*, we mean a category whose membership is determined by a common relational structure rather than by common properties. For instance, for X to be a *bridge*, X must connect two other entities or points; for X to be a *carnivore*, X must eat animals. Relational categories contrast with entity categories such as *tulip* or *camel*, whose members share many intrinsic properties.” Relational categories in this sense have received growing attention (e.g., Asmuth & Gentner, 2017; Gentner & Asmuth, 2019; Goldwater & Schalk, 2016; Jamrozik & Gentner, 2020; Minervino, Margni, & Trench, 2023; Markman & Stilwell, 2001; Steininger, Wittwer, & Voss, 2024). We consider *brace* a relational category (and *brace* as a relational label) because for X to be a brace, it must prevent another part of the structure from moving and, in this way, preserve the stability of the structure.

A third point of clarification concerns the role of language in analogical processing. We have argued that language is a powerful contributor to analogical learning. This raises the question of whether we are claiming that language is *necessary* to engage in analogical processing. The answer is no. Research has shown that even prelinguistic infants (as young as 7 months and even 3 months of age) can carry out comparisons and make analogical generalizations. Further, their profile of results is consistent with the predictions of structure‐mapping theory (Ferry, Hespos, & Gentner, 2015; Hespos, Anderson, & Gentner, 2020). Thus, analogical comparison appears to be present in infants well before the advent of language. In the current study, children in the HA condition were able to acquire the idea of bracing even without hearing the label, consistent with the idea that language is not *necessary* for analogical learning. However, as we have emphasized above, there is also considerable evidence that comparison and language are mutually scaffolding in learning.

### 4.7 Implications for education

Our findings here join with others in showing that comparison and structural alignment can help learners grasp new concepts. The children in our study mostly did not begin with the idea of a diagonal brace. But with a brief exposure, children given highly alignable models grasped the idea well enough to transfer it to new structures even after a few days’ delay. Of course, we would not claim that the children in our study fully understood the point of the diagonal brace—namely, that the triangle is a stable polygon, so incorporating triangles lends stability to a structure. But their experience of using the diagonal to make a building strong may provide an entry point that opens the way to further learning.

Our findings also dovetail with other work indicating that comparison can be a highly effective technique for teaching STEM and other domains (Alfieri, Nokes‐Malach, & Schunn, 2013; Begolli & Richland, 2016; Clement, 1993.; Gadgil, Nokes‐Malach, & Chi, 2012; Goldwater & Gentner, 2015; Honomichl & Chen, 2006; Jee & Anggoro, 2019; Kurtz et al., 2001; Mason, 2004; Matlen, Vosniadou, Jee, & Ptouchkina, 2011; Mix, Smith, & Crespo, 2019; Rittle‐Johnson & Star, 2009; Thompson & Opfer, 2010; Vendetti, Matlen, Richland, & Bunge, 2015). For example, Richland and McDonough (2010) found that college students learned the concepts of permutation and combination better when comparisons were supported during instructions (i.e., by providing comparative gestures and visibly aligning problem cases) than when comparisons were made difficult. Similarly, Jee and Anggoro (2019) found that scaffolding comparisons and highlighting correspondences helped third graders understand the day/night cycle. The current studies add to this literature by showing how contrastive language and comparison can benefit science learning. These findings have important implications for STEM education. Indeed, it has been suggested that research on comparison offers a critical framework for insights into educational practices, and a bridge through which practitioners can facilitate students’ learning of challenging classroom concepts (Alfieri et al., 2013; Goldwater & Schalk, 2016; Jee et al., 2010; Richland & Simms, 2015; Vendetti et al., 2015).

## 5 Conclusion

The current studies show that analogical comparison and language can help children gain understanding of a challenging spatial concept—the diagonal brace. It further provides evidence for a novel way in which language can combine with comparison to facilitate relational learning—namely, that linguistic contrast can invite contrastive comparison. Our findings show that these effects can occur even without deliberate teaching. Our findings join other work supporting the claim that language and culture can provide representational resources that scaffold relational cognition. We suggest that the joint action of language and comparison may be critical in explaining children's rapid learning in the spatial domain and beyond.

## Conflict of interest

The authors report no conflicts of interest.

## Ethical approval statement

The study was approved by the Office of the Institutional Review Board Administration at Northwestern University, Evanston.

## Supporting information



Supporting information

## Data Availability

The data reported in this article are available through the Open Science Framework at https://osf.io/ye7w5/?view_only=0e0f7534840048d4826e32cfa080a4be

## Notes

1 Typically, spatial relational terms involve a relationship between two things—a figure and a ground. For example, one object (the *figure*) could be *inside* or *behind or left of* another object (the *ground*). In contrast, the *midpoint* relation requires understanding that the figure lies between *two* ground objects, and that it is equidistant from both. Thus, this relation is expected to be more challenging than simpler figure‐ground relations, all else being equal.

2 The initial and test rooms had furniture in the same configuration, with objects that matched by category—for example, bed→bed—but that were dissimilar in appearance. Object dissimilarity creates difficulty in mapping between model rooms (Marzolf & DeLoache, 1994; DeLoache & Sharon, 2005).

3 This is the idea of *progressive alignment*, in which successfully carrying out a close comparison can result in a more abstract representation, which then facilitates the processing of more abstract relational comparisons (Kotovsky & Gentner, 1996; Haryu, Imai, & Okada, 2011).

4 In another study, Loewenstein and Gentner (2005, Experiment 4) gave 3‐ and 4‐year‐old children a more challenging mapping task (a cross‐mapping task, in which surface matches were inconsistent with the relational mapping) and compared two sets of spatial terms: the systematic set, *top, middle, bottom* and the less systematic set, *on, in, under*. As expected, 3‐ and 4‐year‐olds performed better with the systematic set.

5 This finding argues against the view that comparison and association tap into the same underlying process, as suggested by Bassok and Medin (1997). See Honke and Kurtz (2019; Honke, Kurtz, & Laszlo, 2020) for further evidence that different processes are involved in comparison versus association.

6 By *contrastive comparison*, we do not mean to convey a different kind of comparison process. Our assumption is that the same structural alignment and mapping process takes place as in other comparisons, with the distinction that rather than focusing on commonalities in the output, the learner will focus on differences.

7 The term *brace* is relational in that it refers to a functional part of a construction: specifically, a brace prevents another component from moving and in this way preserves the stability of a structure. (We amplify this point in the General Discussion.)

8 The correct term should be “stable,” but we used “strong” as a more familiar word to children.

9 The effect size of the logistic models was estimated in terms of standardized odds ratio using the “standardize_parameters” function in the “effectsize” package (Ben‐Shachar et al., 2021). It indicates an increase of *b* in odds (of using a brace) associated with a 1 unit change in the standardized predictor.

10 Importantly, the training manipulations seem to benefit girls as well as boys. Across the two studies, there was a consistent trend for both boys and girls in the high‐alignment condition to outperform those in the low‐alignment condition, and for those receiving the contrastive label to outperform those receiving control language (although not all the results reach significance). This suggests that our training is applicable to a broad set of prior skills and that it can potentially improve both boys’ and girls’ performance.

11 In SME (the Structure‐mapping Engine), the process model of structure‐mapping theory, common structure, and alignable differences are automatically produced by the mapping engine (Forbus, Ferguson, Lovett, & Gentner, 2017).

12 The homesigners were children whose hearing losses prevented them from learning a spoken language, and who had not been exposed to a conventional sign language. Such children often develop their own gestural language, which their parents may also use (Goldin‐Meadow, 2003).

13 The two samples were matched on general cognitive and perceptual abilities and came from the same middle‐class social stratum. A prior vocabulary elicitation task had shown that homesigners (using gestures) were as likely as hearing children to express objects and actions; however, they were far less likely to express spatial relations, such as *in, on, under, top*, and *bottom*.

14 In an investigation of the effects of late sign language exposure, Karadöller et al. (2022) showed that neither late language exposure nor the frequency and the type of language used by the learners predicted their spatial memory accuracy.
