# Supplementary material for: Structural Alignment and Linguistic Contrast Help Children Learn a Key Principle of Spatial Construction
Source: Cogn Sci. 2025 Dec 25;49(12):e70149. doi: 10.1111/cogs.70149 (PMC12741211; doi:10.1111/cogs.70149)
Supplement: Supplementary file 1 — Supporting information [file COGS-49-e70149-s001.docx]

**Additional Analysis of the Interaction between Alignment and Language**

Besides the Chi-square analysis reported in the main text, we conducted two additional analyses to explore the interaction between alignment and language: (1) by including the interaction term in the regression models, and (2) through nonparametric bootstrapping.

The regression results suggest that including the interaction term might cause the problem of unstable standard errors (possibly due to multicollinearity) and in some cases, render the models uninterpretable. Table S1 contrasts the current regression outputs with those reported in Tables 1 (for Experiment 1) and 2 (for Experiment 2) in the main text. (We also report standard errors here for a clear comparison.)

Table S1: Comparison of regression results before and after including the interaction term

(*b* stands for regression coefficient; *se* stands for standard error; and *p* stands for p-value)

|  |  | Before including interaction  (same as in Table 1) | |  | After including interaction | | |
| --- | --- | --- | --- | --- | --- | --- | --- |
|  |  | Alignment | Language |  | Alignment | Language | Interaction |
| E1 | Transfer | *b* = 1.938  *se* = 0.695  *p* = .005 | *b* = 2.278  *se* = 0.717  *p* = .001 |  | *b* = 17.5  *se* = 1631  *p* = .99 | *b* = 17.8  *se* = 1631  *p* = .99 | *b* = -15.9  *se* = 1631  *p* = .99 |
|  |  | Before including interaction  (same as in Table 2) | |  | After including interaction | | |
| E2 | D1 Repair | *b* = 1.172  *se* = 0.410  *p* = .004 | *b* = .877  *se* = 0.408  *p* = .032 |  | *b* = 1.625  *se* = 0.655  *p* = .013 | *b* = 1.340  *se* = .661  *p* = .043 | *b* = -0.784  *se* = 0.847  *p* = .35 |
|  | D2 Repair | *b* = 1.803  *se* = 0.467  *p* = .000 | *b* = 1.128  *se* = 0.451  *p* = .012 |  | *b* = 2.110  *se* = 0.827  *p* = .011 | *b* = 1.458  *se* = 0.852  *p* = .087 | *b* = -0.473  *se* = 1.009  *p* = .64 |
|  | Near Transfer | *b* = 2.976  *se* = 0.691  *p* = .000 | *b* = 1.877  *se* = 0.562  *p* = .001 |  | *b* = 17.4  *se* = 1233  *p* = .99 | *b* = 16.4  *se* = 1233  *p* = .99 | *b* = -14.7  *se* = 1233  *p* = .99 |
|  | Far Transfer (Construction) | *b* = 0.605  *se* = 0.421  *p* = .15 | *b* = 0.941  *se* = 0.425  *p* = .027 |  | *b* = 1.034  *se* = 0.557  *p* = .064 | *b* = 1.413  *se* = 0.590  *p* = .017 | *b* = -1.034  *se* = 0.855  *p* = .23 |
|  | Far Transfer (Recognition) | *b* = 0.960  *se* = 0.410  *p* = .019 | *b* = 1.109  *se* = 0.411  *p* = .007 |  | *b* = 1.340  *se* = 0.661  *p* = .043 | *b* = 1.484  *se* = 0.657  *p* = .024 | *b* = -0.643  *se* = 0.849  *p* = .45 |

We also ran a nonparametric bootstrapping analysis to examine this interaction. In bootstrapping, the data were sampled with replacement N times (where N = the sample size of conditions; 16 for E1 and 29 for E2), and a score was computed for this new sample to quantify the magnitude of the interaction. Given that we expected labels to diminish the gap between high- and low-alignment, the score was computed as a difference of difference, where we first subtracted the number of LA children who passed the tasks from that of HA children (as in the main text) and further subtracted this value of the Label condition from that of the Control. This score was expected to be positive, indicating a larger alignment effect in the Control than in the Label conditions. This procedure was repeated 1000 times, resulting in 1000 observations of this critical score. Significance is determined by checking whether its 95% confidence interval excludes zero. As shown in Table S2, out of all measures, the only significant score was found for D2 Near Transfer task in E2, where the difference between high- and low-alignment was significantly larger in the Control condition than in the Label condition. This finding converges with the Chi-square analysis in the main text and suggests a lack of consistent interaction effect.

Table S2. The results of bootstrapping analysis for the interaction effect

|  | Task Measure | 95% Confidence Interval |
| --- | --- | --- |
| E1 | Transfer | [-4, 8] |
| E2 | D1 Repair | [-12, 6] |
|  | D2 Repair | [-8, 11] |
|  | D2 Near Transfer | [2, 17]* |
|  | D2 Far Transfer (Construction) | [-16, 2] |
|  | D2 Far Transfer (Recognition) | [-13, 8] |

**E2: Other Exploratory Measures**

In Experiment 2, we included three further exploratory questions at the end of Day 2 to assess the strength of learning—specifically, we asked children, “*Why do you think it’s* [the braced building] *strong?*”, “*Can you point to what makes that one strong?*”, and (for the Label condition) “*What do we call this?*”. For the first *why* question, we coded whether children mentioned the idea of diagonal brace^[[1]](#footnote-1)^; for the second pointing question, we coded whether children correctly pointed to the diagonal brace in the stable training building; for the last question, we coded whether children spontaneously spelled out the word *brace*. The results are summarized in Table S3. Fisher’s Exact tests revealed that across language, the High Alignment condition was more likely than the Low Alignment condition to allude to the idea of bracing in their explanations and pointing to the diagonal brace. Alignment was not significantly related to whether children recalled the brace label.

Table S3. E2: The number of children who answered the end-of-study exploratory questions correctly (the max number in each cell is N=29).

|  |  |  |  | |
| --- | --- | --- | --- | --- |
| **Question** | **Alignment/Language** |  | HA | LA |
|  |  |  |  |  |
| *“Why do you think it’s strong?”* | Label |  | 24 | 13 |
|  | Control |  | 24 | 10 |
|  |  |  |  |  |
| *“Can you point to what makes that one strong?”* | Label |  | 28 | 21 |
|  | Control |  | 29 | 17 |
|  |  |  |  |  |
| *“What do we call this?”* | Label |  | 12 | 8 |
|  | Control |  | NA | NA |
|  |  |  |  |  |

1. Correct responses to the *why* question included 1) saying any of the following words: *diagonal*, *brace, x, cross, triangle,* or *upward/ angled line*, 2) gesturing the diagonal, or 3) pointing to the diagonal brace in the model building. [↑](#footnote-ref-1)
